# The moderating effect of gender on physical activity participation and physical fitness in children

**DOI:** 10.4102/hsag.v29i0.2672

**Published:** 2024-08-20

**Authors:** Howard Gomwe, Lesego Phiri, Chioneso Show Marange

**Affiliations:** 1 Skills Centre, Faculty of Health Sciences, Sefako Makgatho Health Sciences University, Pretoria, South Africa; 2 Department of Statistics, Faculty of Science and Agriculture, University of Fort Hare, East London, South Africa

**Keywords:** physical activity, physical fitness, gender, school learner, children, moderating

## Abstract

**Background:**

The level, direction or presence of the relationship between perceived physical activity (PA) participation and physical fitness (PF) in children may differ by gender and this will assist in designing gender-specific interventions to promote PA participation, which in turn improves PF levels.

**Aim:**

This study aimed to establish the moderating effect of gender on the relationship between perceived PA participation and PF.

**Method:**

A cross-sectional study was conducted with a cohort of 870 primary school children aged 9–14 years old. Perceived PA participation was measured using the PAQ-C questionnaire while PF levels were measured using cardiorespiratory endurance (i.e. VO2 max).

**Results:**

The results showed significant gender differences for perceived PA participation levels (*t* = −3.40, *p* ≤ 0.001) and PF (*t* = −11.59, *p* ≤ 0.001), with boys reporting higher levels than the girls. Perceived PA participation had a positive, weak and statistically significant correlation with PF (*r =* 0.251; *p* ≤ 0.001). Gender significantly moderates the relationship between perceived PA participation and PF (β_3_ = 3.518; 95% confidence interval [CI]: 0.642 to 6.395; *p =* 0.017).

**Conclusion:**

The moderating effect of gender on the relationship between perceived PA participation and PF highlights the differences in physiology as well as the societal PA associated roles played by boys and girls.

**Contribution:**

The study has established that the relationship between perceived PA participation and PF is more pronounced among boys than girls.

## Introduction

Physical activity (PA) and physical fitness (PF) play a critical role in the lives of children. It is commonly believed that children who participate in PA will have a higher level of PF and that the relationship is causal. To examine the relationship between these two concepts, it is perhaps best to start by defining them. The terms PA and PF are frequently used interchangeably because they are closely related. Physical activity is defined as any form of bodily movement, planned or unplanned, produced by skeletal muscles that require the use of energy (Tian et al. 2021). On the other hand, PF is the ability of the body to perform successfully and competently, to be healthy and resistant to disease, to take pleasure in leisure activities, and to adapt to various situations (Dong et al. 2020). Physical fitness comes in two main varieties: skill-related PF, which includes power, balance, agility, coordination, reaction time and speed. The other form is health-related PF, defined by cardiovascular fitness, body composition, physical strength, muscular endurance, flexibility and body awareness (Godana & Enyew 2021). Generally, PA leads to PF, which is well known for preventing cardiovascular diseases (American College of Sports et al. 2018; Menescardi et al. 2021).

The level of PA participation in children impacts their health and development throughout their life span (World Health Organization [WHO] 2020). Furthermore, physical inactivity is strongly associated with major non-communicable diseases (NCDs). Several studies demonstrated that PA lowers the risk of NCDs such as coronary heart disease, high blood pressure, stroke, metabolic syndrome and type II diabetes (American College of Sports et al. 2018; Pavlović et al. 2022; WHO 2020). Low level of PA is the fourth largest risk factor for mortality (WHO 2020). Furthermore, a lack of PA participation has been highlighted as one of the major risk factors for the global disease burden, accounting for an estimated 1.4 million deaths in 2016 and an 18.4% rise since 2006 (Shu et al. 2024). To address the required levels of PA participation for NCDs prevention, the WHO produced worldwide PA guidelines. According to the WHO, children and adolescents should accumulate moderate-to-vigorous PA (MVPA) at least 60 min per day (WHO 2020).

Gender differences in PA participation have been shown to exist in previous research conducted worldwide, with lower PA participation observed in girls (Palacios-Cartagena et al. 2022). This is also supported by a recent study conducted by Shao and Zhou (2023), who assessed the influential factors of PA habits for school children in Malaysia. The results indicated that boys performed more PA habits than girls. The existing gender differences may result from the societal roles that boys and girls perform (Gomwe, Phiri & Marange 2023). Generally, girls are less physically active and have been reported to participate less in sports than boys (Gomwe et al. 2022). In addition, girls tend to receive less social support to engage in PA (Menescardi & Estevan 2021), which results in less enjoyment in PA participation (Gomwe et al. 2023). Biological reasons may also contribute to the differences in PA participation between boys and girls. Some studies have reported that sexual maturity is a possible explanation for these differences (McCarthy & Warne 2022) such that, lower PA participation levels in girls may be because of maturing earlier than boys. However, a study conducted in Norway to examine PA participation and PF levels among 13- to 15-year-old children revealed that more girls participate in PA than boys (Rullestad, Meland & Mildestvedt 2021).

Marta et al. (2012) assessed the differences between PF among boys and girls in Portugal. This cross-sectional study involved 312 children between 6 and 10 years old. The PF assessment included sets for aerobic fitness, strength, flexibility, speed, agility and balance. Boys outperformed girls in all tests except for balance and flexibility. The explanation is because of differences in hormone levels, muscle mass, body composition and growth trends. There are also differences in heart size and efficiency in utilising oxygen. Amusa et al. (2011) assessed health-related PF among rural primary school children in Tshannda rural area in South Africa. The results showed that the boys generally performed higher than the girls. However, girls were superior to boys in the tests of flexibility. Their study hypothesised that South Africa’s socioeconomic change since 1994 has led to a decrease in children’s PA and fitness levels. They reported that black rural children were disadvantaged in terms of PA facilities.

Literature has shown a direct relationship between the level of PA participation and PF in children (Palacios-Cartagena et al. 2022). For example, in a study conducted in China, Tian et al. (2021) reported a positive correlation between perceived PA participation and PF of 715 school children, both boys and girls, in Yangling District. The results showed that the positive correlation between perceived PA participation and PF was more pronounced in rural areas than urban settings. Most published work and scientific evidence reveal that PA participation is a predictor of PF levels (Chen et al. 2021; Palou et al. 2019). That is, given higher levels of PA participation, an increase in the levels of PF is anticipated. This is also the case for lower PA participation levels, which results in low PF levels. That is, low levels of PA participation could be an underlying cause of the decrease in PF levels; hence, the study of PA participation and PF is vital in designing health promotion intervention to improve the general well-being, especially in children and adolescents (Nakagawa et al. 2020). Thus, PA participation along with PF plays a crucial role in the general well-being of individuals and their relationship and interaction are major areas of research in public health (López-Gil et al. 2020).

Generally, in children and adolescents, PA improves PF, that is, cardiorespiratory and muscular fitness. Several studies have reported gender differences in PA participation or perceived PA participation as well as PF among children. In addition, various researchers have also reported the relationship that exists between PA participation or perceived PA participation and PF in children (Gea-García et al. 2020). It is, therefore, imperative to establish whether the level, direction, or presence of the relationship between perceived PA participation and PF in children differs by gender. Thus, while perceived PA participation can predict PF in children, this relationship may be stronger, weaker, or non-existent for a specific gender as compared to the other. Thus, in such a case, gender is regarded as a moderating variable. This study will focus on a cohort of South African rural primary school children to determine if the gender of participants moderates the relationship between perceived PA participation and PF. This will help to better inform various stakeholders in designing gender-specific interventions to encourage PA participation in children, hence improving their PF levels that will, in turn, prevent NCDs later in their adult lives.

### Hypotheses

The following alternative hypotheses were formulated.

**H1_A_:** There exist statistically significant differences in levels of perceived physical activity participation by gender of participants.

**H2_A_:** There exist statistically significant differences in levels of physical fitness by gender of participants.

**H3_A_:** There is a significant correlation between perceived physical activity participation and physical fitness.

**H4_A_:** Gender significantly moderates the relationship between perceived physical activity participation and physical fitness.

## Methods

### Study design and participants

The study utilised a cross-sectional design using a cohort of primary school children aged 9–14 years old in the Eastern Cape province, South Africa.

### Study setting and sampling

The research study was conducted in three selected municipalities in the Eastern Cape province of South Africa. Firstly, out of the six provincial districts in the Eastern Cape province, one metro district municipality (Amathole municipality) was conveniently sampled, and two district municipalities were purposively sampled (Chris Hani municipality and Oliver Tambo municipality). That is, the metro district municipality was chosen by prioritising urban settings closer to the hosting institution, while the two district municipalities were purposively chosen because of the vast number of rural primary schools within these districts. Secondly, a random selection was made from a list of schools in quintiles 1, 2 and 3 supplied by each municipality’s district education departments. The South African Department of Education categorised public schools using a method known as quintile ranking to provide financial resources. The poorest primary schools are in quintile one, while the least deprived are in quintile five. This study only covered schools that were poor and which do not charge tuition fees. Thirdly, eighteen primary schools were randomly selected using computer-generated software. They included six rural schools from Chris Hani municipality, six urban schools from Amathole municipality, and six rural schools from Oliver Tambo municipality. The last step involved randomly selecting a 10th of the participants from each chosen school using the class teachers’ registers. The research study excluded all learners who were handicapped and those who were sick. The study included *n* = 870 participants, that is, 176 from urban areas, 235 from peri-urban areas, and 459 from rural schools.

### Measurements

Two data collection methods were utilised: a survey and an actual PF exercise to measure VO2 max. For the survey, demographic variables such as gender (boy or girl), age (in years) and place of residence (urban, peri-urban and rural) were collected. Furthermore, to measure perceived PA participation, we used the Physical Activity Questionnaire for Older Children (PAQ-C). Generally, the questionnaire measures the perceived PA levels in children aged 8–14 years during a normal school week in a year (i.e. the actual amount of PA in the last 7 days). This questionnaire has been validated in a similar diverse population of primary school learners in South Africa (Van Biljon et al. 2018). A pilot survey was conducted using a convenient sample of primary school learners from the same age group and similar demographic profiles before the main data collection. Before the pilot study, changes were made to the original PAQ-C questionnaire, especially regarding replacing sporting activities. For instance, cross-country skiing, ice hockey and badminton were replaced with soccer, athletics, and rugby to suit the sporting activities that the local schools provide. The main purpose of the pilot survey was to enhance the clarity of the terminology used and the applicability and availability of the sporting activities listed in the questionnaire. According to Kowalski, Crocker and Donen (2004), we categorised the mean score of the PAQ-C as low (score of 1.00–2.33), moderate (score of 2.34–3.66) or high (score of 3.67–5.00) PA levels. Lastly, PF was measured using VO2 max, which is cardiorespiratory fitness. VO2 max was assessed by a validated maximal multistage 20-m shuttle run test. The 20-m shuttle run test predicts maximal aerobic capacity and involves 23 levels, where a level is a series of 20-m shuttle runs. Each level lasts 60 s and the time between the recorded ‘bleeps’ decreases for each new level. The starting speed is normally 8.5 km/h and then increases by 0.5 km/h with each new level. School learners were familiarised with the procedure first, and the results were entered as the number of laps per level taken to complete the 20-m shuttle run test.

### Statistical analysis

Data analysis was conducted using SPSS version 29. A descriptive analysis was conducted on the general demographic characteristics of the participants. The independent sample *t*-test was used to test for the mean differences of the study’s theoretical variables by respondents’ gender. Pearson’s correlation coefficient was used to establish the existing linear relationships between perceived PA participation and PF. To perform the moderation analysis, gender (a categorical variable) was first converted to a dummy variable by taking two possible values of 0 and 1 (Girls – 0; Boys – 1). After that, the interaction variable corresponding to the multiplicative term between perceived PA participation and the moderator variable (gender) was created. To determine the significance of the moderation effect of gender, a multiple linear regression model using the Hayes process macro in SPSS (Hayes 2017) was examined. Utilising the 95% confidence intervals (CI) with 10 000 replications, the significance of the interaction effect of gender and perceived PA participation was used to determine the moderating effect. Finally, an interaction plot was plotted to better understand and visualise the moderating findings.

### Ethical considerations

Ethical clearance was obtained from the institutional research ethics committee of the University of Fort Hare. Approval was also granted by the respective education district offices and the Department of Health to conduct the study in the primary schools. Parental consent was obtained for all the participating learners because they were all minors (below the age of 18 years). Participation was voluntary, and the collected data were kept confidential and anonymous. Informed consent was obtained from the participants in this study.

## Results

### Demographics

The sample consisted of *n* = 870 primary school learners, with girls (*n* = 519, 59.7%) constituting the majority of the sample. The learners were from 9 to 14 years of age, with an average age of 11.20 ± 1.60 years. Most of the participants were from rural areas (*n* = 459, 52.8%), followed by those from peri-urban areas (*n* = 235, 27.0%) and 20.2% (*n* = 176) from urban areas.

### Hypotheses testing

#### Hypotheses 1 and 2

The independent sample *t*-test examined gender differences in perceived PA participation and PF levels. Levene’s test for homogeneity of variance was used. This test verified that the assumption of equal variances holds only in the perceived PA participation sample. Therefore, equal variances were only assumed in this sample and not in the PF sample. Using the relevant output (see Table 1), the results showed significant gender differences for perceived PA participation levels (*t =* −3.40, *p* ≤ 0.001), with boys (mean = 2.39 ± 0.44) having higher levels than the girls (mean = 2.29 ± 0.42). This finding supports the first alternative hypothesis suggesting that, in general, boys have high levels of perceived PA participation as compared to girls. Regarding the second hypothesis, results showed significant gender differences for PF (*t =* −11.59, *p* ≤ 0.001), with boys also reporting higher PF levels than girls. There is sufficient statistical evidence to conclude that statistically significant differences exist in levels of PF by gender of the selected primary school children.

**TABLE 1:** Descriptive summary and independent samples *t*-test for the gender differences in perceived physical activity participation and physical fitness.

Variables	Combined	Females	Males	*t*	*p*
	Mean ± SD	Mean ± SD	Mean ± SD		
Perceived PA participation	2.33 ± 0.43	2.29 ± 0.42	2.39 ± 0.44	-3.40	< 0.001*
PF	31.88 ± 10.23	28.64 ± 8.05	36.68 ± 11.20 0.83	-11.59	< 0.001*

SD, standard deviation; PA, physical activity; PF, physical fitness.

* , statistically significant differences.

#### Hypothesis 3

The Pearson’s correlation coefficient was used to examine the strength, direction and nature of the relationship between perceived PA participation and PF. The cut-off points proposed by Cohen (1988) were used to determine the strength of the established linear relationships. Thus, Cohen (1988) classifies the value of the correlation coefficient into three categories: weak (0.10 to 0.29), moderate (0.30 to 0.49) and strong (0.50 to 1.00). In Table 2, the results of the correlation analysis show that the correlation between perceived PA participation and PF was positive, weak and statistically significant (*r =* 0.251; *p* ≤ 0.001). This result supports the third alternative hypothesis. Thus, a significant correlation exists between perceived PA participation and PF, meaning that those with higher perceived PA participation had increased PF.

**TABLE 2:** Pearson’s correlation coefficients for the relationship between perceived physical activity participation and physical fitness.

Variables	PF
Perceived PA participation	*r* = 0.251
-	*p* ≤ 0.001**

*r*, Pearson’s correlation coefficient; PA, physical activity; PF, physical fitness.

** , Correlation is significant at the 0.01 level (2-tailed).

#### Hypothesis 4

**FIGURE 1:**
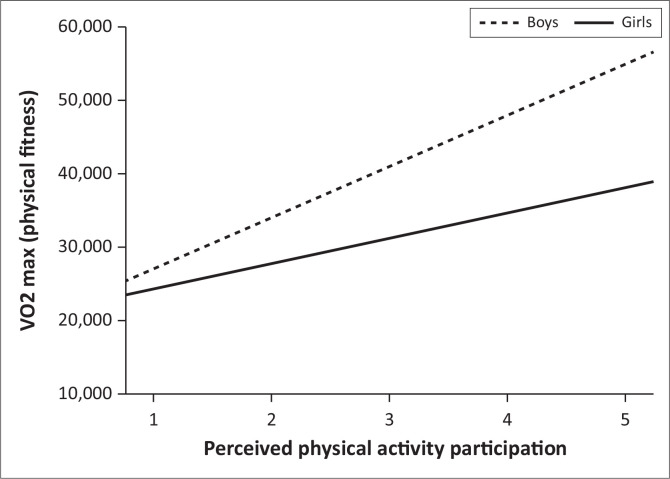
Interaction plot for the moderating effect of gender on the relationship between perceived physical activity participation and physical fitness.

Table 3 shows the output of the established multiple linear regression model to examine the moderating effect of gender on the relationship between perceived PA participation and PF. The regression beta path estimates revealed that the interaction effect was positive and statistically significant (β_3_ = 3.518; 95% CI [0.642 to 6.395]; *p =* 0.017). This shows that gender significantly moderates the relationship between perceived PA participation and PF. The conditional effects (see Table 4) reveal that in the category of boys, perceived PA participation has more of an effect on the model than in the category of girls. The interaction effect and the associated plot (see Figure 1) reveal that for both genders, individuals presenting high levels of perceived PA participation exhibit a positive relationship with PF (i.e. higher levels of perceived PA participation are associated with higher levels of PF). This association is more pronounced among boys; thus, as the levels of perceived PA participation increase, boys showed a more evident association with PF. Thus, gender significantly moderates the relationship between perceived PA participation and PF.

**TABLE 3:** Hayes process macro multiple linear regression beta estimates to determine the moderating role of gender on the relationship between perceived physical activity participation and physical fitness.

Direct effects on PF (Y)	Beta estimates		95% confidence intervals		*t*	*p*
	Estimate	SE	Lower	Upper		
Constant	20.753	2.238	16.360	25.147	9.272	< 0.0001*
Perceived PA participation (X)	3.441	0.961	1.555	5.327	3.580	0.0004*
Gender (M)	-0.718	3.501	-7.589	6.154	-0.205	0.838
Interaction effect (Perceived PA participation *Gender)	3.518	1.466	0.642	6.395	2.401	0.017*

* , Statistically significant effects.

SE is the standard error for the respective estimates. 95% confidence intervals using 10 000 replications are presented.

Independent variable (X): Physical activity (PA).

Dependent variable (Y): Physical fitness (PF).

Moderator variable (M): Gender.

**TABLE 4:** Conditional effects of perceived physical activity participation at different categories of gender.

Gender	Effect to the model		*t*	*p*	95% confidence intervals	
	Effect	SE			LLCI	ULCI
0 (Girls)	3.441	0.961	3.580	< 0.0001	1.555	5.327
1 (Boys)	6.959	1.107	6.289	< 0.0001	4.787	9.131

SE, standard error; LLCI, lower limit confidence interval; ULCI, upper limit confidence interval.

## Discussion

This study looked at the moderating effect of gender on the relationship between perceived PA participation and PF among primary school learners in South Africa. Our findings showed that boys have higher levels of perceived PA participation and PF than girls. These results support those reported in previous studies where PA participation has been shown to be lower in girls (Palacios-Cartagena et al. 2022; Shao & Zhou 2023). In addition to the biological differences between boys and girls, the observed differences in our study may be because girls are more confined to performing indoor household chores, while boys concentrate on various outdoor activities (Gomwe et al. 2023). Furthermore, Gomwe et al. (2022) reported the PA and sedentary behaviour of the same cohort of primary school learners. They presented that girls were more sedentary than boys, and this might explain why girls had lower PA and PF than boys.

Furthermore, this study’s findings also revealed a positive correlation between perceived PA participation and PF. Thus, the higher the perceived PA participation among primary school children, the higher their levels of PF. Literature has also reported the existence of a direct relationship between PA participation and PF in children (Palacios-Cartagena et al. 2022; Tian et al. 2021). Our findings showed a weak correlation between perceived PA participation and PF, which is in support of Gea-García et al. (2020) who reported a weak relationship between PA and PF among learners aged between 11 and 14 years. Because this study used VO2 max to measure PF, the possible reason for the findings is that regular PA participation causes physiological changes in the body, such as an increase in cardiorespiratory endurance. Thus, regular exercise improves blood circulation, which leads to cardiovascular fitness and is vital in improving health, especially in children and adolescents (Nakagawa et al. 2020; Palou et al. 2019; Riso et al. 2019).

Our major finding was that gender has a significant moderating effect on the relationship between perceived PA participation and PF among primary school children in South Africa. Both boys and girls presenting high levels of perceived PA participation exhibit a positive relationship with PF (i.e. higher levels of perceived PA participation are associated with higher levels of PF). This association is more pronounced among boys; that is, as the levels of perceived PA participation increased, boys showed a more evident association with PF than girls. According to several studies, boys are superior to girls in aerobic fitness, which is mainly linked to cardiac size and oxygen carrying (Amusa et al. 2011). Therefore, with a similar amount of exercise, boys tend to gain more cardiovascular fitness than girls.

## Conclusion

Adolescence is a critical stage in developing PA patterns that stretch to adulthood. Therefore, examining the gender differences that exist in PA participation and PF levels is essential among primary school children as this can be used to design gender-specific interventions earlier to avoid NCDs later in life. The study recommends reintroducing physical education to the South African primary school curriculum. The use of theory-based interventions and longitudinal studies (Heeren et al. 2018) might aid to reveal the barriers faced by girls in PA participation. Future research should use actual PA participation instead of perceived PA participation, which is subjective.

### Limitation

In this study, PA participation was measured as a perceived variable, which might not account for the actual PA participation of the learners. Although perceived PA participation has been widely used to measure PA participation, this might be considered a limitation of this study.
