# Corrigendum: The moderating effect of gender on physical activity participation and physical fitness in children

**DOI:** 10.4102/hsag.v29i0.2853

**Published:** 2024-09-19

**Authors:** Howard Gomwe, Lesego Phiri, Chioneso Show Marange

**Affiliations:** 1 Skills Centre, Faculty of Health Sciences, Sefako Makgatho Health Sciences University, Pretoria, South Africa; 2 Department of Statistics, Faculty of Science and Agriculture, University of Fort Hare, East London, South Africa

In the published article, Gomwe, H., Phiri, L. & Marange, C.S., 2024, ‘Corrigendum: The moderating effect of gender on physical activity participation and physical fitness in children’, *Health SA Gesondheid* 29, a2672. https://doi.org/10.4102/hsag.v29i0.2672, the article section was incorrectly allocated.

Instead of:

Review Article

It should be:

Original Research

The authors apologise for this error. The correction does not change the study’s findings and significance, the overall interpretation of the study’s results, or the scientific conclusions of the article in any way.

